# Case of Spinal Irritation

**Published:** 1860-06

**Authors:** C. D. Henton

**Affiliations:** Myersville


ARTICLE 3.

### REPORT OF A CASE OF SPINAL IRRITATION.

BY C. D. HENTON, M. D., OF MYERSVILLE.

Was called, May 25th, 1856, to see Miss E. W.

Found a young lady, aged 18, of a pale, bloodless appearance, in bed, surrounded by the friends and neighbors, from whom I received the following history:

That she had been confined to her bed for four weeks, that during all this time she had had "*Spells*," which, within the last 48 hours, had been increasing both in severity and duration. During this time she had been attended by an irregular, whose diagnosis was, "inactivity of the secreting and excreting organs, and spasmodic affection of diaphram."—All I could learn of the previous treatment was, that a great variety of remedies had been used, and among them, that "Sampson of the drug store," calomel—which I readily believed, as she was now decidedly ptyalized. I also learned that her catamenia had first appeared about one year since, but had been very irregular, both as to time and quantity.

A paroxysm soon came on, which was characterised by violent muscular disturbance, the ordinary expression of countenance was lost and replaced by a vacant stare; at short intervals she threw her limbs about convulsively, beat her breast, tore her hair, and frequently urrered short, inarticulate sounds, which might not inaptly be compared to the baying of a small dog.

A careful examination of the case, both during the fit and also during the interval, the absence of emaciation, and there being throughout the paroxysm, the "appearnce as of a concealed will," also being great tenderness upon pressure over spinous processes of last cervical and upper three dorsal vertebra, forced the conviction upon me that it was only a form of that protean malady, hysteria, dependent on spinal irritation. I therefore directed my remedial agents accordingly, and, suffice to say, that under the use of opium, quinine, iron, valerian, &c. internally, and the "unguentum antimonii," externally, over tender vertebra, I had the satisfaction of seeing my patient *rapidly* and permanently recover; and now, nearly four years since,. is in possesion of perfect health.

